# Editorial: Breeding for intercropping, volume II

**DOI:** 10.3389/fpls.2026.1924989

**Published:** 2026-07-21

**Authors:** Pierre Hohmann, Martin Weih, Diego Rubiales

**Affiliations:** 1 Department of Biology, Health and Environment, Faculty of Pharmacy and Food Sciences, University of Barcelona, Barcelona, Spain; 2 Department of Crop Production Ecology, Swedish University of Agricultural Sciences, Uppsala, Sweden; 3 Institute for Sustainable Agriculture, Consejo Superior de Investigaciones Científicas (CSIC), Córdoba, Spain

**Keywords:** agroecology, breeding for mixtures, crop diversification, GxExM interactions, mixing ability, participatory breeding, variety testing

## From opportunity to implementation

Intercropping is gaining renewed attention as agriculture seeks systems that are more resilient, resource-efficient and ecologically functional. By combining crop species or genotypes in the same field, intercropping can improve yield stability, resource capture, weed and disease regulation, and biodiversity. Yet a major bottleneck remains: most crop varieties are still bred, tested and registered for monocultures. Farmers are therefore encouraged to diversify their cropping systems while often lacking varieties specifically selected for performance in mixtures.

This Research Topic, *Breeding for Intercropping - Volume II*, builds on the first volume by shifting the focus from the potential of intercropping towards its implementation in breeding, testing and agricultural practice. The four contributions in this volume address complementary parts of this challenge: adapting breeding schemes, defining intercrop-relevant traits, broadening the crop portfolio for diversified systems, and rethinking variety testing.

## Adapting breeding strategies

Dubey et al. ask a central practical question: can existing monocrop breeding strategies be adapted for intercrops, or do breeders need entirely new programmes? Their study shows that the answer depends on the relationship between monocrop and intercrop performance. When this relationship is strong, early selection in monoculture may still provide useful information. When it is weak, however, intercrop testing must be introduced earlier, because monocrop performance becomes a poor proxy for mixture performance.

This is an important contribution because it moves the discussion beyond a simple contrast between monocrop and intercrop breeding. Instead, it provides a more strategic view: breeders can decide when and how to introduce mixture testing depending on trait heritability, genetic correlations and available resources. Such transition pathways are essential if breeding for intercropping is to become feasible beyond a small number of specialised programmes.

## Traits, ideotypes and interaction

Abou Khater et al. bring the discussion to cool-season food legumes, using faba bean - durum wheat intercropping as a case study. Their work highlights that good intercrop varieties cannot be defined by sole-crop yield alone. In mixtures, plants must not only produce well, but also interact productively with their companion crop.

The authors identify traits such as combined grain yield, 100-seed weight, number of pods per plant and canopy height as relevant for faba bean performance in intercropping. These traits reflect both productivity and compatibility. They also underline a broader principle: breeding for intercropping requires ideotypes designed around interaction. A successful intercrop variety is not necessarily the most competitive or the highest-yielding in pure stand, but the genotype that contributes most to the performance, stability and manageability of the whole crop mixture.

## Expanding the crop portfolio

Ghidoli et al. focus on camelina, an underutilised oilseed crop with potential value in rotations, cover cropping and diversified systems. Although their study does not directly test intercropping performance, it addresses a key upstream requirement: diversified agriculture needs suitable crop germplasm.

Through genetic characterisation and bulk selection, the authors developed a new camelina line combining early maturity, increased thousand-seed weight and high oil content. These traits are relevant for practical integration into more complex cropping systems. Early maturity can improve compatibility with short growing windows or relay systems, larger seed size may facilitate sowing and harvest, and high oil content strengthens the crop’s economic value. The study therefore reminds us that breeding for intercropping is not only about improving major crop combinations. It also depends on expanding the range of species and varieties available for diversification.

## From breeding to variety testing

Hohmann et al. widen the perspective from breeding methods to the institutions that determine whether mixture-adapted varieties reach farmers. They argue that current variety testing and registration systems remain largely oriented towards monocultures. As a result, breeders have little incentive to select for mixing ability, and farmers receive limited information on which varieties perform well in intercrops.

The article proposes practical ways forward: mixture sub-trials, standard tester varieties, trait-based and genomic predictors, on-farm testing networks, open data infrastructures and policy incentives. Importantly, this does not require replacing existing variety testing systems. Rather, it suggests complementing them so that diversified agriculture becomes a recognised target environment. Treating mixing ability as a sustainability-relevant trait could help connect breeding innovation, agroecological policy and farmer decision-making.

## Shared messages from volume II

Together, the four articles show that intercropping performance is an interaction phenotype. It depends on the genotype of the focal crop, the companion crop, the environment and the management system. This complexity makes breeding more challenging, but not impossible. Concepts such as general and specific mixing ability, trait-based proxies, genomic prediction and multi-environment testing can help breeders organise this complexity into workable selection strategies.

The volume also highlights the need for pragmatic transition steps. Fully dedicated intercrop breeding programmes may be ideal in some cases, but many breeding organisations need lower-threshold approaches. These may include adding mixture testing at selected stages, using standard companion varieties, identifying traits linked to intercrop performance, developing germplasm for minor or emerging crops, and connecting breeding trials with participatory farm networks. Finally, breeding alone is not enough. Mixture-adapted varieties will only matter if farmers can access seed, compare variety combinations, receive reliable advice and operate within policy and market environments that reward diversification. Breeding for intercropping is therefore both a biological and institutional challenge.

## Outlook

The next phase of research should integrate breeding, agronomy, ecology and policy more closely. We need more empirical data on genetic correlations between monocrop and intercrop performance, better phenotyping protocols for interaction traits, models to prioritise promising variety combinations, and prediction tools trained on mixture-specific data. Living labs, participatory trials and open data platforms can help capture real-world genotype x environment x management interactions.

Breeding for intercropping challenges a quiet assumption of modern crop improvement: that varieties should be judged primarily by how well they perform in pure stands. Diversified agriculture asks a different question: which genotype performs well with others, under which conditions, and for which system-level goals?

The articles in this second volume show that the field is ready to address this question. They point towards a shift from monocrop optimisation to interaction-aware breeding, from isolated yield evaluation to system performance, and from experimental intercropping to institutional support for crop diversification.

